# Natural Product Research in the Australian Marine Invertebrate *Dicathais orbita*

**DOI:** 10.3390/md11041370

**Published:** 2013-04-23

**Authors:** Kirsten Benkendorff

**Affiliations:** Marine Ecology Research Center, School of Environment, Science and Engineering, Southern Cross University, PO Box 157, Lismore, NSW 2480, Australia; E-Mail: Kirsten.benkendorff@scu.edu.au; Tel.: +61-2-66203755; Fax: +61-2-66212669

**Keywords:** bioactivity, biosynthesis, brominated secondary metabolites, choline ester, indole

## Abstract

The predatory marine gastropod *Dicathais orbita* has been the subject of a significant amount of biological and chemical research over the past five decades. Natural products research on *D. orbita* includes the isolation and identification of brominated indoles and choline esters as precursors of Tyrian purple, as well as the synthesis of structural analogues, bioactivity testing, biodistributional and biosynthetic studies. Here I also report on how well these compounds conform to Lipinski’s rule of five for druglikeness and their predicted receptor binding and enzyme inhibitor activity. The composition of mycosporine-like amino acids, fatty acids and sterols has also been described in the egg masses of *D. orbita*. The combination of bioactive compounds produced by *D. orbita* is of interest for further studies in chemical ecology, as well as for future nutraceutical development. Biological insights into the life history of this species, as well as ongoing research on the gene expression, microbial symbionts and biosynthetic capabilities, should facilitate sustainable production of the bioactive compounds. Knowledge of the phylogeny of *D. orbita* provides an excellent platform for novel research into the evolution of brominated secondary metabolites in marine molluscs. The range of polarities in the brominated indoles produced by *D. orbita* has also provided an effective model system used to develop a new method for biodistributional studies. The well characterized suite of chemical reactions that generate Tyrian purple, coupled with an in depth knowledge of the ecology, anatomy and genetics of *D. orbita* provide a good foundation for ongoing natural products research.

## 1. Introduction

*Dicathais orbita*, commonly known as the Australian Dogwhelk or Cartrut shell, is a predatory marine gastropod in the family Muricidae. This family of marine molluscs is well known for the production of the ancient dye Tyrian purple [1,2], which was the first marine natural product to be structurally elucidated by Friedlander in 1909 [3]. Over a century later, there remain major gaps in our knowledge of the ecological role and biosynthesis of this secondary metabolite [4,5]. However, significant progress has been made by Australian researchers over the last five decades [1,6,7,8,9,10,11,12,13,14,15], thus providing a foundation for using *D. orbita* as model species in natural products research.

**Figure 1:**
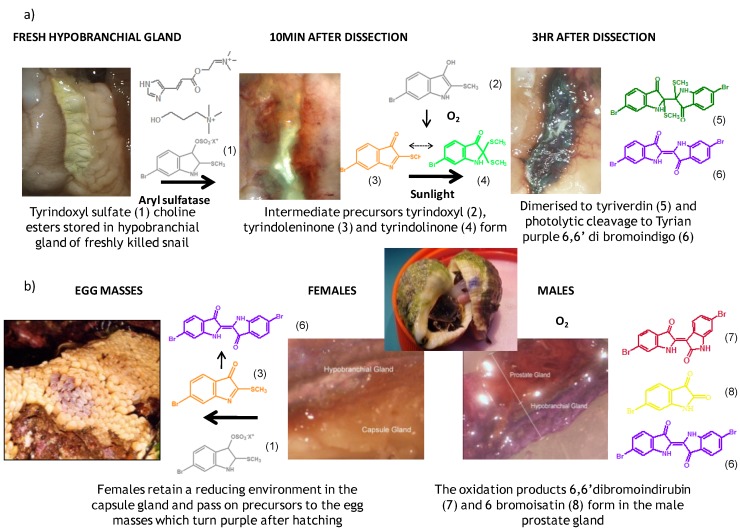
(**a**) The development of Tyrian purple in the hypobranchial gland of *Dicathais orbita*; (**b**) The transfer of reduced precursors from the capsule gland of females to the egg capsules and the oxidation of precursors in the prostate gland of male *D. orbita*.

As a common and relatively large gastropod on rocky intertidal reefs, *Dicathais orbita* is an important educational resource and has been the focus of study by a wide diversity of Australian postgraduate research students. Investigations into the natural products of *D. orbita* first commenced with the Ph.D. thesis of Joe Baker in 1967 [9], who established the ultimate precursors of Tyrian purple from the biosynthetic organ, the hypobranchial gland (e.g., Figure 1). This work was continued in the Ph.D. thesis of Colin Duke [16], who identified the intermediate precursors and synthesized a range of structural analogues. After a twenty year gap, my Ph.D. study into the antimicrobial properties of Australian molluskan egg masses identified the precursors of Tyrian purple from *D. orbita* as interesting lead compounds for bioactivity studies [17]. This initiated an ongoing program of research focused on *D. orbita* and their bioactive compounds, resulting in the completion of a further four Ph.D.s [18,19,20,21], one Masters of Biotechnology [22] and eight Honors theses [23,24,25,26,27,28,29,30], with an additional five Ph.D.s currently in progress. 

Around the same time as the research on *D. orbita* natural products chemistry commenced, Australia research students began investigating the ecology and life history of this species. The first in-depth study into the biology of *D. (aegrota) orbita* was undertaken by Bruce Phillips in Western Australia, whose Ph.D. thesis was published in 1968 [31]. Several additional student theses investigating the life history and ecology of *D. orbita* have been recently undertaken in South Australia [24,28,29]. *Dicathais (Thais) orbita* was also the major focus of a Ph.D. thesis by Gibson investigating imposex caused by TBT pollution on the east coast of Australia [32]. This established *D. orbita* as one of the first Australian invertebrate model species for ecotoxicology and an important indicator for environmental monitoring [32]. *D. orbita* was also included in the Ph.D. thesis of well known Australian ecologist Peter Fairweather, who investigated interactions between predators and prey on intertidal shores [33]. *D. orbita* has been subsequently included as a model species in several other student theses investigating environmental stressors and human impacts [34,35]. These insights into the ecology and life history of *D. orbita* have greatly facilitated ongoing natural products research, through interesting biological insights and population assessments, which help ensure sustainable collection.

To be suitable as a model system for innovative natural products chemistry research, a wealth of biological data is required on the organism, along with extensive familiarity with secondary metabolism system to be studied. *Dicathais orbita* is a candidate model species for the biosynthesis of brominated indoles, as these natural products and the associated biosynthetic glands in this marine mollusk are relatively well known (Figure 1). Useful biological traits for the selection of model species also include availability and life history features that make them easy to manipulate and maintain in the laboratory, as well as genetic knowledge and potential economic benefit [36]. Indeed *D. orbita* is a relatively large, long-lived gastropod that is common on rocky reefs in temperature Australian waters [33,37,38,39] and it also occurs as a pest predator on some molluskan aquaculture farms [40]. This species produces benthic egg capsules that each contain thousands of embryos that can be studied through several stages of larval development [41] and the reproductive cycle and anatomy of the adults is well documented [15,42,43]. *D. orbita* is resilient to environmental fluctuations [pers. obs] and both broodstock and juveniles can be easily maintained in laboratory aquaria [44]. The taxonomy of this species is well resolved [45], as is its systematic position within the Rapaninae subfamily of Muricidae [46] and the Gastropoda [47,48,49] more broadly. Genetic information on this species is also accumulating [5,50], with preliminary genome sequencing currently underway. A significant transcriptome database exists for a related species of *Rapaninae* [51]. As highlighted by Rittschof, and McClellan-Green [36], the power of model organisms could increase exponentially with input from multidisciplinary research teams that work from the molecular level, through the various levels of biological organization, to the ecosystem level. The combination of natural products chemistry and biological research undertaken on *D. orbita* to date establishes this species as potentially useful model for future studies on the evolution and biosynthesis of marine secondary metabolites, as well as for new method development e.g., [52].

## 2. Secondary Metabolites from *Dicathais orbita*

### 2.1. Brominated Indole Derivatives

The hypobranchial gland of muricid mollusks is the source of the ancient dye Tyrian purple, for which the main pigment is well established to be a brominated derivative of indole, 6,6 dibromoindigo (**6**, Figure 1) [1,2,3]. Original observations of the hypobranchial glands confirmed that the dye pigment itself is not present in the live mollusk, but rather is generated after a series of enzymatic, oxidative and photolytic reactions. In 1685, Cole [53] first described the changes in the hypobranchial glands of muricid mollusks, from a white fluid to yellow, through various shades of green and blue, before obtaining the final purple color after exposure to sunlight. This series of color reactions was also noted by Baker [1,8,9] in the hypobranchial glands from the Australian species *D. orbita*; illustrated in Figure 1. The indole precursors span a range of chemical properties (Table 1a) from the water soluble salt of tyrindoxyl sulfate (M.W. 337, 339, log *p* < −0.3) to the highly insoluble tyriverdin (M.W. 514, 516, 518, log *p* > 4.6).

Baker and Sutherland [8] first isolated a salt of tyrindoxyl sulphate (**1**, Figure 1) from an ethanol extract of the hypobranchial gland of *D. orbita* and identified this as the ultimate precursor to the dye Tyrian purple. They also isolated an enzyme with sulfatase activity capable of hydrolyzing tyrindoxyl sulfate and initiating the production of Tyrian purple by exposure to sunlight [8]. Baker and Duke [6,7,10,11] subsequently isolated and identified the intermediate precursors tyrindoxyl (**2**) and tyrindoleninone (6-bromo-2-methylthio-3*H*-indol-3-one) (**3**), as well as tyrindolinone (**4**), a methanethiol adduct of tyrindoleninone (Figure 1a). Using various organic solvents, Baker and Sutherland were also able to isolate a yellow light insensitive compound identified as 6-bromoisatin, and the immediate precursor to Tyrian purple, a green light sensitive compound tyriverdin [8]. The structure of tyriverdin (**5**, Figure 1) was subsequently corrected by Christophersen *et al.* [54] as an indole dimer that forms spontaneously from the reaction between tyrindoxyl and tyrindoleninone (Figure 1a). 6-Bromoisatin (**8**, Figure 1) is considered to be an oxidation artifact in this sequence of reactions [2,8] and is a precursor of the red Tyrian purple isomer 6,6′-dibromoindirubin (**7**) [55]. These oxidation products do occur naturally in small amounts of the extracts from males, but were not detected in female *D. orbita* hyprobranchial gland and gonad extracts (Figure 1b), suggesting sex specific differences in the chemical environment of these glands [13].

Table 1Molecular properties of (**A**) brominated indoles and (**B**) choline esters isolated from *Dicathais orbita* using Molinspiration Cheminformatics (2012). Molecular weight for Br^79^ isotopes.

**Table:** (**A**)

Compound	MW/Formula	Log *p*^a^	Polar surface area/volume	No. non-H atoms	No. H bond acceptors ^b^	No. H bond donors ^c^	Rotatable bonds	No. rule of 5 violations ^d^
Tyrindoxyl sulfate 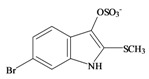	337.196 C_9_H_7_BrNO_4_S_2_^−^	−0.346	82.224/211.287	17	5	1	3	0
Tyrindoxyl 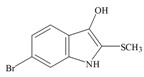	258.14 C_9_H_8_BrNOS	3.375	36.019/173.614	13	2	2	1	0
6 Bromoisatin 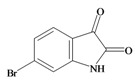	226.029 C_8_H_4_BrNOS	1.615	49.933/141.457	12	3	1	0	0
Tyrindoleninone 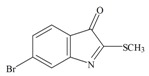	256.124 C_9_H_6_BrNOS	2.889	29.963/168.021	13	2	0	1	0
Tyrindolinone 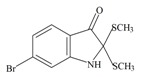	304.234 C_10_H_10_BrNOS_2_	2.999	29.098/208.356	15	2	1	2	0
Tyriverdin 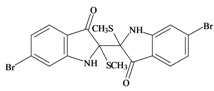	514.264 C_18_H_14_Br_2_N_2_O_2_S_2_	4.66	58.196/334.697	26	4	2	3	1
Tyrian purple 6,6′ dibromoindigo 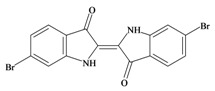	420.06 C_16_H_8_Br_2_N_2_O_2_	4.47	65.724/259.728	22	4	2	0	0
6,6′ Dibromoindirubin 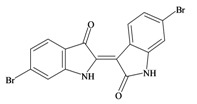	420.06 C_16_H_8_Br_2_N_2_O_2_	4.47	65.724/259.728	22	4	2	0	0

**Table:** (**B**)

Compound	MW/Formula	Log *p*^a^	Polar surface area/volume	No. non-H atoms	No. H bond acceptors ^b^	No. H bond donors ^c^	Rotatable bonds	No. rule of 5 violations ^d^
Murexine 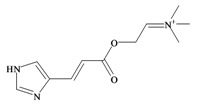	224.284 C_11_H_18_N_3_O_2_^+^	−3.373	54.988/219.763	16	5	1	5	0
Senecoiycholine 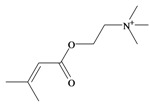	186.275 C_10_H_20_NO_2_^+^	−2.096	26.305/200.647	13	3	0	5	0
Tigloylcholine 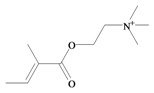	186.275 C_10_H_20_NO_2_^+^	−2.33	26.305/200.647	13	3	0	5	0
Choline 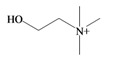	104.173 C_5_H_14_NO^+^	−4.236	20.228/120.158	7	2	1	2	0

^a^ Log *p* is based on octanol-water partition coefficient; ^b^ H bond acceptors include O & N atoms; ^c^ H bond donors include OH and NH groups; ^d^ Rule of 5 violations are based on the molecular descriptors used by Lipinski *et al.* [56] for “drug-like” molecules (log *p* ≤ 5, molecular weight ≤500, number of hydrogen bond acceptors ≤10, and number of hydrogen bond donors ≤5).

An interesting point of difference in *D. orbita* indole chemistry, relative to other Muricidae, is the production of a single brominated ultimate precursor molecule [2,8,57]. Four prochromogens including brominated and nonbrominated indoxyl sulfates have been suggested for *Murex brandaris* [58], which then generate a mixture of purple 6,6 dibromoindigo, as well as blue indigo and monobromoindigo [2]. Baker [1] also demonstrated the complexity of purple precursors obtained from the hypobranchial glands of some other Muricidae species. These Tyrian purple precursors are also transferred to the egg masses of *D. orbita* (Figure 1b) and other Muricidae mollusks [12,59]. Similar to the hypobranchial glands, the egg masses of other Muricidae were found to contain a more complex mixture of brominated and non brominated indole, as well as other brominated compounds including imidazoles, quinolones and quinoxalines [17,60,61]. Consequently, the Australian species *D. orbita* appears to be a particularly pure source of 6,6′ dibromoindigo and the simplicity of the single precursor make it a good model for biosynthetic studies of brominated indoles. On the other hand, the diversity of indoles and brominated compounds in the Muricidae family more broadly provides a good opportunity for phylogenetic investigations into the evolution of secondary metabolism.

### 2.2. Choline Esters

In 1976, Baker and Duke made an important breakthrough when they isolated choline from the hypobranchial glands of *D. orbita* and demonstrated that tyrindoxyl sulfate is stored as a choline ester salt [7]. This salt is hydrolysed by an arylsulphatase enzyme, which is also stored within the hypobranchial gland [8], to generate the intermediate precursors of Tyrian purple (Figure 1a). Both choline, and to a lesser extent murexine (β-imidazolyl-4(5)acrylcholine) (Table 1b) were found to be associated with tyrindoxyl sulfate [7]. *N*-Methylmurexine was also suggested to be present in the hypobranchial gland extracts [7], but this was subsequently questioned by Duke *et al*. [62,63]. 

In 1996, Roseghini *et al*. [64] reported a survey of choline esters and biogenic amines from the hypobranchial glands of 55 species of gastropods. *Dicathais* (*Neothais*) *orbita* was found to contain significant quantities of murexine and senecioylcholine (Table 1b). Dihydromurexine was the dominant choline ester found in some other Muricidae species, but was not detected in *D. orbita* [64]. Shiomi *et al.* [65] have also identified tigloylcholine (Table 1b) in other muricids from the genus *Thais*. These authors pointed out that senecioylcholine is a structural isomer of tigloylcholine and since senecioylcholine was only previously identified by thin layer chromatography and is indistinguishable from tigloylcholine using this method, it may have been misidentified in the earlier studies [65]. 

### 2.3. Mycosporine-Like Amino Acids, Fatty Acids and Sterols in the Egg Masses

In addition to reports on the indole derivatives in *D. orbita* egg masses [60,66], the composition of mycosporine-like amino acids (MAAs) and fatty acids has been documented for this species. MAAs are small sunscreening compounds with an absorption maxima of 310–360 nm [67]. They are produced via the shikimate pathway in algae, fungi and bacteria, but animals, including marine invertebrates, are thought to acquire these secondary metabolites through diet or symbiosis [67,68]. Przeslawski *et al*. [69] revealed that mycosporine-glycine and shinorine were the dominant MAAs in *D. orbita*, along with porphyra-334 and mycosporine-2-glycine and trace amounts of palythine. Mycosporine-taurine, palythene, asterina-330 and palythinol were not detected in this species, although an additional unknown peak with an absorption maxima of λ 307 nm was reported in *D. orbita*, along with two other Muricidae [69]. The composition of MAAs was found to be strongly influenced by phylogeny in molluskan egg masses, but not by the adult diet or levels of UV exposure in the spawning habitat [69]. This suggests that predatory marine mollusks, such as *D. orbita*, are able to bioaccumulate MAAs from their prey and transfer these into the egg masses to protect their developing embryos. Higher MAA concentrations were found in *D. orbita* egg masses with viable embryos in comparison to inviable egg masses [69]. The inviable eggs of *D. orbita* typically appear pink or purple in color, as opposed to the usual yellow color [59], thus indicating further chemical changes, likely due to the photolytic degradation of Tyrian purple precursors. By absorbing UV radiation in normally developing Muricidae egg masses, MAAs may play an essential role in maintaining the bioactive indole precursors prior to larval hatching. Alternatively, by absorbing in the UV spectra [13,27], the brominated indoles may provide further protection against harmful UV rays.

In a comparative study of lipophylic extracts of the egg masses from a range of molluskan species, Benkendorff *et al*. [70] revealed that *D. orbita* egg capsules predominately contain palmitic and stearic acid. Unlike many other gastropod egg masses, no unsaturated fatty acids were found in the leathery egg capsules of *D. orbita* and related neogastropods [70]. The extracts from *D. orbita* egg masses contained a large amount of sterol, predominately cholesterol, but with smaller amounts of cholestadienol, cholestanol, methyl cholestadienol and methylcholestenol [70]. No cholestadiene or stigmatenone were found, although some unknown sterols were detected. It is unclear why Neogastropoda with leathery egg capsules, such as *D. orbita*, have a much higher saturated fatty acid and sterol content than gastropods with gelatinous egg masses, although the later may require unsaturated fatty acids to maintain fluidity in the gelatinous matrix. 

## 3. Bioactivity of *Dicathais orbita* Extracts and Compounds

### 3.1. Drug-Likeness of *D. orbita* Secondary Metabolites

Using the online chemoinformatics software Molinspiration (version 2011.06) the drug-likeness (Table 1) and bioactivity scores (Table 2) are predicted for the main secondary metabolites from *D. orbita*. Drug-likeness is based on Lipinskis “Rule of 5” [69], which considers whether various molecular properties and structure features of a particular molecule are similar to known drugs. These properties, such as hydrophobicity, electronic distribution, hydrogen bonding characteristics, molecule size and flexibility (Table 2), influence the bioavailability, transport properties, affinity to proteins, reactivity, toxicity, metabolic stability of the molecule and thus potential for use as a pharmaceutical drug. Of all the indole derivatives examined (Table 1a), only a single violation of the rule of 5 was found. This was for tyriverdin, due to a molecule weight exceeding 500 mass units (Table 1a). As expected, choline and all of the choline esters conform to the rule of 5 for drug-likeness (Table 1b). 

Table 2Bioactivity of (**A**) brominated indoles and (**B**) choline esters isolated from *Dicathais orbita* based on calculated distribution of activity scores from Molinspiration (version 2011.06) ^#^, as well as known bioactivity from the published literature.

**Table:** (**A**)

Compound	GPCR ligand	Ion channel modulator	Kinase inhibitor	Nuclear receptor ligand	Protease inhibitor	Enzyme inhibitor	Other known bioactivity
Tyrindoxyl sulfate 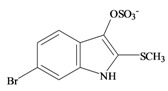	0.22 *	0.02	−0.13	−0.36	0.10	0.73 **	-
Tyrindoxyl 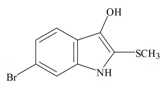	−0.56	−0.09	−0.41	−0.71	−1.00	−0.11	Unstable in O_2_
6 Bromoisatin 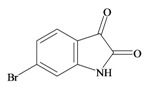	−1.08	−0.49	−0.50	−1.62	−1.07	−0.39	Anticancer, induces apoptosis, anti-bacterial [12,71]
Tyrindoleninone 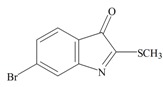	−0.93	−0.39	−0.69	−1.16	−1.15	−0.43	Anticancer, induces apoptosis, anti-bacterial [12,71]
Tyrindolinone 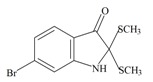	−0.87	−0.54	−0.89	−1.03	−0.93	−0.51	Unstable in O_2_
Tyriverdin 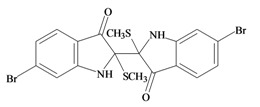	−0.23	−0.23	−0.29	−0.34	−0.17	−0.17	Bacteriostatic, inhibits FDA hydrolysis [12]
Tyrian purple 6,6′ Dibromoindigo 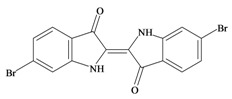	−0.32	−0.30	0.22 *	−0.05	−0.36	−0.01	Highly insoluble, no apparent antibacterial or anticancer activity [4]
6,6′ Dibromoindirubin 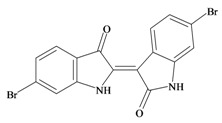	−0.78	−0.74	0.45 *	−0.28	−0.61	0.01	GSK-3 inhibitor [72]

**Table:** (**B**)

Compound	GPCR ligand	Ion channel modulator	Kinase inhibitor	Nuclear receptor ligand	Protease inhibitor	Enzyme inhibitor	Other known bioactivity
Murexine 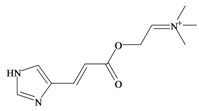	0.38 *	0.50 *	−0.16	−1.70	−0.36	0.84 **	Neuromuscular blocking and nicotinic action. No muscarinic effects. Paralysis of the skeletal musculature, toxic to mice at high doses (i.v. LD_50_ 8.5 mg/kg, s.c. LD_50_ = 50 mg/kg); human clinical dose (EC_50_ = 1 mg/kg) [63,73]
Senecoiycholine 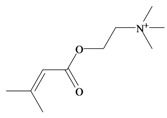	−0.39	0.33 *	−1.04	−1.28	−0.95	0.35 *	Neuromuscular blocking and nicotinic action. No muscarinic effects [63]
Tigloylcholine 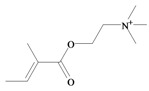	−0.45	0.32 *	−1.37	−1.31	−1.35	0.41 *	Toxic to mice (i.v. LD_50_ = 0.92 mg/kg) [64]
Choline 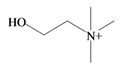	−2.64	−2.21	−3.84	−4.93	−3.94	−2.18	Essential nutrient, precursor for the neurotransmitter acetyl choline [74]

^#^ Larger value bioactivity scores indicate a higher probability that the molecule will be active; * potential activity; ** high potential activity.

The drug-likeness for Tyrian purple maybe over-estimated. The log *p* values for 6,6-dibromoindigo is perhaps lower than anticipated considering the fact that this compound is highly non-polar and generally insoluble at room temperature in all organic solvents [2]. Tyrian purple can only be extracted out of tissue or cloth using hot (>100 °C) DMF or DMSO. It appears to form dimers or higher polymers due to the van der Waals attraction between bromine atoms [75], which contribute to the high stability of the compound, but nevertheless the low solubility makes it an unlikely drug candidate. Despite the same log *p* value (Table 1a) 6,6′ dibromoindirubin appears to be slightly more soluble in non-polar solvents at room temperature (pers. obs.), perhaps due to reduced polymer formation in this isomer.

### 3.2. Bioactivity of *D. orbita* Brominated Indoles

The predicted Molinspiration bioactivity scores for *D. orbita* brominated indoles identify the ultimate precursor tyrindoxyl sulfate as the most likely pharmacophore. This compound shows potential as a GPCR ligand and enzyme inhibitor (Table 2a). Unlike the intermediate precursor compounds, this polar brominated indoxyl sulfate salt has not been directly tested for cytotoxicity in antibacterial and anticancer screening assays. This is because bioassay guided fractionation of *D. orbita* extracts has revealed most of the activity in the more lipophilic fractions of chloroform extracts and generally no activity is found in the polar methanol water fractions [12,19,26,71,76,77], where tyrindoxyl sulfate is mostly concentrated. Nevertheless, tyrindoxyl sulfate has been present in some of the anticancer extracts showing bioactivity against MCF-7 breast cancer cells *in vitro* [26] and against DNA damaged colon cells *in vivo* [78] and could contribute to the observed activity. Tyrindoxyl sulfate is likely to be metabolized and transported differently to the other less polar compounds *in vivo* (Table 1a). This, along with the predicted enzyme binding activity, suggests that tyrindoxyl sulfate might be worthy of further bioactivity studies.

At the other extreme of polarity (Table 1a), the Tyrian purple pigments have predicted protein kinase receptor interaction (Table 1b). This predicted activity is supported for 6,6′ dibromoindirubin, which was shown to be a selective GSK-3 inhibitor, but with limited activity against CDK1/Cyclin B or CDK5/p25 [72,79]. The 6,6′ dibromoindigo isomer was not tested in this study and although predicted to have some protein kinase activity (Table 2a), the extreme insolubility of this compound presents problems for bioactivity assessment.

Despite the compatibility with drug-likeness, few of the intermediate brominated indoles from *D. orbita* produced high enough bioactivity scores on Molinspiration to indicate interesting pharmacophores for receptor binding (Table 2a). Nevertheless, purified extracts containing 6-bromoisatin and tyrindoleninone do show broad spectrum antibacterial and anticancer activities [4,12,19,23,71,77,80]. Of particular interest is the >100 fold selective cytotoxicity towards human lymphoma and female reproductive cancer cell lines (KGN, JAr, OVCAR-3), compared to freshly isolated untransformed peripheral blood monocytes and female granulosa cells [19,22,23,71,80]. Furthermore, these brominated indole derivatives appear to induce apoptosis rather than necrosis in the reproductive cancer cell lines, as indicated by caspase 3/7 activity and DNA fragmentation from TUNNEL staining [19,71]. Preliminary work on these brominated indoles using flow cytometry with propidium iodine and annexin staining indicates they also induce apoptosis in lymphoma cells but not in CaCO_2_ colon cancer cells [23,77,81]. However, more recent studies on purified 6-bromoisatin and tyrindoleninine indicate they do induce apoptosis in the H2T9 colon cancer cell line [81]. Furthermore, a rodent model for colon cancer using a concentrated extract containing these two brominated indoles shows that apoptosis is induced *in vivo* and unpublished studies indicate that 6-bromoisatin is the main active factor [81]. The mode of action for these brominated indole derivatives is currently unknown and as they are unlikely to bind with the receptors or enzymes listed in Table 2, further studies are required.

The dimeric compound tyriverdin was not predicted to have any bioactivity based on known pharmacophores for receptor or enzyme binding (Table 2a). Nevertheless, this compound has been identified as a potent bacteriostatic agent against a range of human and marine pathogens, using bioassay guided fractionation of *D. orbita* extracts with the flourescein diacetate hydrolysis antibacterial assay [12]. However, further testing of this compound with alternative methods, such as the standard plate dilution assay [12] or the MTS tetrazolium salt cell proliferation assay [26,27] has failed to confirm the antibacterial activity. Additional procedural controls have indicated that tyriverdin can partially quench the green fluorescence of flourescein in the absence of bacterial cells [27]. However, this quenching did not account for all the apparent reduction in fluorescein absorbance, suggesting that tyriverdin may also interfere with esterase activity or some other mechanism of converting flourescein diacetate to flourescien. However, in addition to violating the molecular weight rule for drug-likeness (Table 1a), tyriverdin also has solubility and instability problems. It is only slightly soluble in some solvents, such as chloroform and dichloromethane, but tends to precipitate out of most solvents (e.g., ether extracts [8] and toluene/hexane [27]), then decomposes to Tyrian purple. This low solubility along with its instability in sunlight and high molecular weight make it an unlikely drug-candidate.

### 3.3. Bioactivity of Choline Esters

Unlike the brominated indole derivatives, the choline esters naturally occurring in *D. orbita* obtained high bioactivity scores in the Molinspiration online chemoinformatics prediction software (Table 2b). In particular, all three choline esters were predicted to inhibit enzymes and modulate ion channels. Murexine, with an imidazole moiety, obtained the highest bioactivity scores and was also the only choline ester predicted to bind to GPCR (Table 2b). The prediction for ion channel modulation is consistent with the known biological activities of these choline esters. Murexine in particular has been thoroughly investigated for toxicity, paralysis of the skeletal musculature, neuromuscular blocking activity and nicotinic action [64,73,82,83] and similar pharmacological properties have been reported for senecioylcholine [64]. Both compounds are almost devoid of muscarinic effects on acetylcholine receptors [64,73]. Murexine was shown to stimulate ganglion, in addition to having depolarizing neuromuscular blocking actions in cat, dog and rat [82]. 

The intravenous LD_50_ for murexine in mice has been established at 6.5 mg/kg [64] to 8.7 mg/kg [73] and death is caused by anoxia secondary to peripheral respiratory arrest [64,73]. Tigloylcholine was estimated to be more toxic, with an i.v. LD_50_ of 0.92 mg/kg in mice [65]. When administered subcutaneously the LD_50_ of murexine in mice was approximately 50 mg/kg and oral delivery was ineffective in doses up to 1 g/kg [64]. Preliminary human clinical trials were conducted with murexine as muscle relaxant on 160 patients. The mean paralysing dose in adult patients was approximately 1 mg/kg i.v. with the paralysis lasting for 3–6 min after a single dose and longer lasting muscular relaxation could be obtained by slow i.v. infusion of a 1/1000 solution of murexine in physiological saline [64,73]. However, murexine caused several side-effects, which were mainly attributable to the nicotinic actions of the drug. 

### 3.4. Antibacterial Activity and Chemical Ecology of the Egg Masses

As part of a screening study on the antimicrobial properties of molluskan egg masses, *Dicathais orbita* was identified as a species of particular interest, with the lipophylic extracts showing strong activity against a range of human and marine bacterial pathogens [66,84]. Bio-guided fractionation identified the brominated indole precursors of Tyrian purple as being responsible for this activity [12]. Based on this activity, Benkendorff *et al*. [12] proposed that defense of the developing embryos against ubiquitous marine pathogens could be the naturally selected role for these brominated indoles in Muricidae evolution. Consistent with this, the surface of the egg capsules of *D. orbita* were found to have very low levels of bacterial biofilm formation, with a high proportion of dead bacteria indicated by live/dead bacterial staining [85]. Using the MTS cell proliferation and broth dilution assay, extracts containing the Tyrian purple precursors from the surface of *D. orbita* egg capsules were effective at inhibiting the growth of the marine biofilm forming bacteria *Pseudoalteromonas* sp. S91, as well as the molluskan pathogen *Vibrio harveyi* [28]. The egg capsules of *D. orbita* were also found to have no protists on the surface and were relatively free of algal fouling compared to other gastropod egg masses [86]. The low surface fouling on these egg masses is likely to be due to a combination of chemical, physical and mechanical defense mechanisms preventing bacterial attachment and persistence on the surface [85].

To investigate whether fatty acids could contribute to the observed antibacterial activity in liphophylic extracts from mollusks [66,76], Benkendorff *et al*. [70] tested a series of lipid mixtures modeled on those found in the egg masses. The lipid mixture modeled on the fatty acid and sterol composition of *D. orbita* and similar Neogastropoda had very limited antibacterial activity against marine pathogens, especially when compared to species with gelatinous egg masses and a high content of polyunsaturated fatty acids [70]. It is possible that the bioactive indoles in *D. orbita* egg masses [12,60] negate the requirement for antimicrobial polyunsaturated fatty acids, or perhaps the transfer of bioactive indoles for defense of the egg masses was selected for due to the absence of alternative secondary metabolites with antibacterial activity in these egg masses. 

### 3.5. Anti-Cancer Extracts, Toxicity & Nutraceutical Potential

Organic extracts from *D. orbita* egg masses, hypobranchial glands and mucus secretion effectively inhibit the proliferation of a range of cancer cell lines [19,23,26,71,77]. Bioassay guided fractionation indicated that the brominated indoles tyrindoleninone and tyrindolinone, as well as 6-bromoisatin are primarily responsible for this activity. A crude chloroform extract containing these brominated indole derivatives has also been shown to stimulate the acute apoptotic response to DNA-damage in the distal colon of mice, thus preventing early stage tumor formation [78]. Unpublished studies from my laboratory on the crude extracts and purified indoles suggest that these have no negative impacts on human immune cell function [22]. The crude extracts are generally not toxic in rodents, but can cause mild idiosyncratic hepatotoxicity in some mice [87]. Nevertheless, some liver damage is common with most chemotherapeutics and *D. orbita* extracts remain of interest due to their selective induction of apoptosis in cancerous or DNA damaged cells [71,78]. Further studies currently underway in my laboratory indicate that purified fractions containing the main active factor 6-bromoisatin have no effect on liver enzymes or hepatocytes *in vivo* [81]. As muricids comprise a traditional component of African [88], European [89], Mediterranean [90] and Asian [91] diets, there is excellent potential for the development of *D. orbita* as a novel medicinal food, particularly for colorectal cancer prevention, due to apparent bioavailability in the gastrointestinal tract. The historical and ongoing consumption of muricid meat implies an absence of symptomatic toxicity, although thorough investigation of the specific bioactive extracts is still required. 

The combination of compounds with a range of bioactivities in the extracts of *D. orbita* is of particular interest for nutraceutical development [40,92]. In addition to the anticancer and antibacterial properties, *D. orbita* extracts appear to have a biphasic effect on progesterone steroidogenesis [19]. Furthermore, indirubin and some indoles are known to have anti-inflammatory properties. 5-Bromoisatin has been patented as an analgesic with sedative properties that reduce bleeding time in mice [93], suggesting 6-bromoisatin in *D. orbita* extracts could also have similar properties. To date, crude extracts from *D. orbita* containing choline esters not have been specifically tested for bioactivity or toxicity, despite the known muscle relaxing activity of these compounds (Table 2b). However, it is logical to assume than a concentrated extract containing these choline esters would retain the associated biological activity. Choline esters have also been suggested to act as immunological adjuvants in combined chemotherapy [94]. An extract containing muscle-relaxing, analgesic properties, antibacterial and anticancer activity could be particularly useful as a nutracetical or medicinal food [40]. Further studies are required to obtain an optimal concentration and combination of compounds to minimize any clinical side effects.

## 4. A Biological Basis for Future Natural Products Research

### 4.1. Biosynthesis of *D. orbita* Brominated Indoles

Basic gaps in our understanding of the gene and protein machinery that underlie Tyrian purple biosynthesis allow for new and exciting discoveries on biohalogenation and methane thiol incorporation into secondary metabolites. Tyrian purple is thought to be synthesized from dietary derived tryptophan in the Muricidae [4,43]. Tryptophan has been detected in the hypobranchial secretory cells of several Muricidae species [14,15]. It is particularly prevalent in the rectum of *D. orbita*, which is embedded in the hypobranchial gland [15,18]. Although it remains unclear how this amino acid is specifically converted into tyrindoxyl sulfate in Muricidae, several biosynthetic enzymes are likely to be involved (Table 3). Tryptophanase is typically involved in converting tryptophan to indole in bacteria, which can then be converted to indoxyl sulphate by a mono- or di-oxygenase enzyme system [95]. The specific enzymes involved in adding methane thiol groups onto the indole ring are unknown, but may involve some sulfur transferase and reductase enzymes (Table 3). Further investigation of these enzymes could uncover novel mechanisms for biotransformation in secondary metabolism.

**Table 3:** Biosynthetic enzymes proposed to be involved in the production of Tyrian purple precursors. The order of enzyme reactions generating the bromo and methylthio derivatives is not known.

Precursor/Substrate	Enzyme	Product
Tryptophan	Trytophanase	Indole
Indole	Dioxygenases	Indoxyl sulfate
Indole/Indoxyl sulfate	Bromoperoxidase	6 Bromoindole/Indoxyl
(6 Bromo) Indoxyl sulfate	Sulfur transferase & Sulfur reductase	(6 Bromo) Methylthio indolone/Tyrindoxyl sulfate
Tyrindoxyl sulfate	Aryl sulfatase	Tyrindoxyl

Specific incorporation of bromine into the 6-position of the indole ring is an unusual feature found in several bioactive marine indoles [96]. Since bromination more ready occurs in the 4 or 7 position, this strongly implies enzymatic bromination during the biosynthesis of tryindoxyl sulfate. Several regiospecific halogenases have been previously identified from bacteria, which are highly substrate specific for tryptophan [97,98]. However, the tryptophan-halogenases reported to date all appear to utilise chlorine over bromine. Jannun and Coe [99,100] reported bromoperoxidase activity in homogenates from hypobranchial glands of *Murex trunculus* and recent histochemical studies by Westley have confirmed the bromoperoxidase activity in *D. orbita* hypobranchial gland tissue [14,18]. A range of bromoperoxidase enzymes have been previously identified from marine algae, bacteria and fungi [101,102], but these do not generally appear to be substrate or regiospecific in their brominating activity. In a preliminary attempt to identify the bromoperoxidase gene from *D. orbita*, Laffy [21] developed primers from consensus sequence regions after multiple sequence alignment of 11 bromoperoxidases available on genebank (4 algal and 3 bacteria). No PCR products were amplified with these primers, despite successful positive controls. This indicates the muricid enzyme shares low sequence conservation at these primer sites or may be a distinct type of brominating enzyme with specificity for 6-bromination of tryptophan/indole for Tyrian purple biosynthesis. Bromination of indole derivatives has been shown to increase their biological activity [80,103] and the identification of novel halogenation strategies will facilitate alternative mechanisms for generating halogenated biologically active molecules for drug development [97].

The conversion of tyrindoxyl sulfate salt to tyrindoxyl and ultimately Tyrian purple requires an aryl sulfatase enzyme [8]. Histochemical studies have confirmed the release of aryl sulfatase on the epithelium of the hypobranchial gland of *D. orbita* [14,18]. Preliminary analysis of the transcriptome from *D. orbita* hypobranchial gland was successful in detecting the aryl sulfatase gene [5,21,50] and full length sequencing has confirmed the molluscan origin of this enzyme [21]. No other biosynthetic genes were identified in this mollusc transcriptome library, although there is good support for a primary role of the hypobranchial gland in protein synthesis, post translational modification and transport [5,21,25,50]. A large number of unidentified sequences were also present in the hyporbanchial gland transcriptome, suggesting possible novel genes, although the suppressive subtractive hybridization technique used only produces short reads, which may have reduced the chance of successful matches to conserved areas of the open reading frames. Nevertheless, there remains a good possibility for the discovery of novel biosynthetic enzymes from *D. orbita*.

### 4.2. Biodistribution of the Secondary Metabolites in *D. orbita*

Knowledge of the anatomical distribution of natural products is essential for understanding the biosynthesis process and optimal methods for extraction. On a basic level, different tissues can be dissected and extracted to determine which produce and/or store the secondary metabolites. This approach was applied to establish the distribution of Tyrian purple pigments and precursor compounds in the male and female reproductive organs of D. orbita [13]. These compounds were found throughout the female pallial gonoduct [13], with significant quantities in the capsule gland, which lies adjacent to the hypobranchial glands, thus providing evidence for maternal investment of these compounds in the egg masses of D. orbita [4,12]. Despite the production of more oxidized compounds in the male prostate gland, relative to the female gonoduct [13], the presence of significant quantities of these brominated compounds in the males suggests that these compounds are not exclusively produced for defense of the egg masses and likely play some role in the adult life history.

Histochemical techniques for proposed biosynthetic constituents can further aid in establishing the primary metabolic origin of natural products and sites of active biosynthesis [14]. Histomorphological properties of biosynthetic tissues may also reveal regulatory mechanisms, modes of transport, storage and secretion, while histological examination can reveal the presence of potential symbionts (see Section 4.3). The hypobranchial glands of D. orbita show remarkable complexity, with seven distinct types of secretory cells located on the epithelial cell surface [15]. At least two cell types appear to be specifically associated with Tyrian purple synthesis. A subepithelial vascular sinus occurs between the hypobranchial gland and gonoduct, surrounding the rectum and rectal gland [15]. However, there appears to be no direct anatomical mechanism for the transfer of precursors to the gonoduct, suggesting that the compounds are independently synthesized in the reproductive organs. This is supported by the presence of bromoperoxidase and aryl sulfatase activity in the female egg capsule gland [14,18].

Histochemical examination of the biosynthetic enzyme activity and precursors in the hypobranchial glands of *D. orbita* by Westley [18] has further revealed that tyrindoxyl sulfate is biosynthesized through the post-translational bromination of dietary-derived tryptophan, within two discrete sites by two distinct modes. Regulated synthesis occurs on the surface of the lateral hypobranchial epithelium, while the subepithelial vascular sinus of the medial hypobranchial gland appears to constitutively synthesize these compounds. Aryl sulfatase is stored in adjacent supportive cells and exocytosis onto the epithelium surface appears to be regulated [18]. The distinct distribution and regulated activity of aryl sulfatase and bromoperoxidase implies *D. orbita* has evolved the capacity to control the release of bioactive indoles and choline esters. This histological evidence provides further support for a naturally selected role of these secondary metabolites in the life history of the mollusc.

More recently, mass spectrometry imaging (MSI) using desorption/Ionization on porous silicon (DIOS) and nanostructured initiator mass spectrometry (NIMS) was applied to examine the biodistribution of secondary metabolites in *D. orbita* tissues [52]. MSI of biological tissues is becoming a popular tool for biodistributional studies of proteins and pharmaceuticals. However, standard Matrix Assisted Laser Desorption/Ionization Mass Spectrometry (MALDI MS) MSI is challenging for secondary metabolites with low molecular weight due to intense matrix signals, interfering with the detection of signals from the less abundant target compounds. Due to the broad range of polarities in the brominated indoles, *D. orbita* hypobranchial gland chemistry proved to be a good model system for “proof of principle” of a new technique involving direct tissue stamping onto porous silicon and NALDI targets [52]. Ongoing research using this technique is providing interesting insights into the distribution of choline esters and changes the secondary metabolite profile over the reproductive season [104]. Mass spectrometry imaging could also be applied to examine the biodistribution of the bioactive compounds in preclinical trials, as previously done using MALDI with pharmaceutical compounds in rodent models [105].

### 4.3. Microbial Symbionts

Tyrian purple, a uniquely marine metabolite, is the brominated derivative of the blue dye indigo, derived from plants in the genus *Isatis* and a range of bacteria [106,107]. This appears to be an interesting case of convergent evolution, although the potential role of bacteria in the production of Tyrian purple precursors is yet to be ruled out. To date it has been assumed that muricid molluscs themselves are responsible for the biosynthesis of Tyrian purple [4]. However, over the last decade there has been increasing recognition for the key role of microbial symbionts in the biosynthesis of marine natural products [108]. The rectal gland, which is embedded in the hypobranchial gland of *D. orbita*, contains an abundant supply of the tryptophan precursor and also appears to be associated with bromoperoxidase activity [14,18]. Bacteria have been observed within specialized invaginations of the rectal gland in the muricid *Nucella lapillus* [109]. The positive identification of biosynthetic bacterial symbionts involved in Tyrian purple precursor production would present a paradigm shift, providing new options for large scale sustainable production of these bioactive metabolites and valuable pigments.

Preliminary attempts to culture the bacteria from *Dicathais orbita* using standard techniques have isolated only one species from the hypobranchial gland and three from the rectal gland, compared to 35 from nonbiosynthetic tissues [30]. The sole bacterium isolated from both of these biosynthetic organs was positive for indole production, suggesting a possible role in Tyrian purple synthesis, although further chemical analysis of the culture supernatant is required. It is also possible that the diversity of bacteria in these biosynthetic organs has been underestimated due to specific environmental requirements for growth. The high concentration of mercaptans, such as dimethyl disulfide, in the hypobranchial gland is likely to create a reducing environment [13]. Furthermore, the production of Tyrian purple precursors in culture must require sufficient bromine ion availability. Therefore, a range of novel culture conditions may be required to facilitate the growth and secondary metabolism of Muricidae symbionts. Considering that by far the majority of microorganisms can not be easily cultured [110], the application of culture techniques alone may not be sufficient to identify the diversity of microbial symbionts in *D. orbita*. Metagenomic-based approaches have provided evidence of a microbial origin for several metabolites produced by marine invertebrates [108] and have been successfully applied to the identification of indigo producing bacterial strains in soil [107].

Recent histological and genetic studies have also revealed the presence of ciliate protozoans within the hypobranchial glands of *D. orbita* [21,50]. These ciliates are most likely feeding on bacteria on the epithelial surfaces and interstitial spaces. At present, it is unclear whether these ciliates are pathogens, symbionts or just facultative opportunists. The ciliates do not seem to be directly involved in the production of Tyrian purple based on a lack of histological correlation in the location of the ciliates [21], compared to the biosynthetic enzymes and precursor compounds [18]. However, the abundance of the ciliates does increase towards the reproductive season [21], which correlates with an increase in biosynthetic activity and indole precursor storage prior to spawning [4,12,18,84]. This suggests that the brominated indole precursors could be involved in regulating the activity and/or abundance of ciliates in *D. orbita*. The secondary metabolites from *D. orbita* have not yet been tested for anti-protozoan activity, however a number of other indoles are known to possess anti-parasitic activity [96]. 

### 4.4. Sustainable Supply

Tyrian purple is the world’s most expensive colorant (1 g = 2439.50 EUR) [111], and is currently extracted from *Purpura lapillus* (10,000 adult snails for 1 g) and South American Muricidae considered at risk from over fishing [89]. The bioactive properties of the brominated indole precursors and the potential for nutraceutical development from the bioactive extracts, provides a further incentive for large-scale sustainable supply. Ecological and life history studies on *D. orbita* [24,31,33,37,38,39,41,112] contribute to our ability to effectively monitor the population size and recruitment potential of this species. In fact *D. orbita* has been used as a model species for estimating population size [113,114] and for monitoring TBT pollution in the Australian marine environment [32,115,116]. However, as top invertebrate predators, Muricidae molluscs are susceptible to population crashes and the persistence of imposex in some populations further increases their susceptibility to over harvest.

Some progress has been made towards the larval culture [41] and sea-based polyculture of *D. orbita* on abalone farms [40]. However, it has not yet been possible to close the life cycle of this species due to the long planktotrophic (feeding) larval stage and lack of known cues for settlement and metamorphosis [41,117]. Nevertheless, progress has been made towards understanding the growth rates and dietary preferences of the juvenile snails [44,118]. Furthermore, Noble *et al*. [112] have established that it is possible to obtain the bioactive indole precursors from a mucus secretion of *D. orbita*, which offers the potential for non-lethal harvest. 

Although generally not suitable for nutraceuticals, chemical synthesis of bioactive metabolites is generally the preferred option for pharmaceutical supply [119]. This can be efficiently achieved for 6-bromoisatin [20,80] and the choline esters [16,62]. These well known molecules can not be patented, but nevertheless provide interesting leads for the chemical synthesis of a range of structural analogues [16,72,79,80,103], thus permitting the assessment of structure activity relationships. Some bioactive marine metabolites are too difficult or expensive to chemically synthesize and previous attempts to chemically synthesis the anticancer precursor of Tyrian purple, tyrindoleninone, have been unsuccessful [16,20]. This is partly due to nonspecific bromination favoring the 5 or 7 position on the indole ring, thus generating low yields for 6-bromoindole derivatives. However, a greater problem occurs in relation to the addition of a methane thiol group at position 2, due to uncontrollable rapid oxidation to 6-bromoisatin. Consequently, tyrindoleninone is not optimal for pharmaceutical development, and holds better potential for human health applications if incorporated into nutraceutical extracts.

The identification of biosynthetic bacteria, enzymes and gene clusters involved in Tyrian purple production could have important implications for application in sustainable production of *D. orbita* brominated indole derivatives, as well as the bioengineering of novel compounds through recombinant expression. Identification of bacterial symbionts that can produce tyrindoxyl sulfate would facilitate the large scale sustainable production of bioactive brominated indoles and Tyrian purple, assuming these bacteria can be cultured. Over the last decade, there have been increasingly frequent reports of gene clusters or gene cassettes for the biosynthesis of marine natural products [108]. Identification of the full gene cluster associated with tyrindoxyl sulphate biosynthesis in Muricidae would open up the potential for recombinant expression of the entire pathway in an heterologous host. This could also facilitate the rational engineering of new metabolites using combinations of enzymes from distinct biosynthetic pathways, which is an important goal for future drug development [98].

## 5. Conclusions

The Australian Muricidae *D. orbita* biosynthesizes a range of biologically active secondary metabolites, which have stimulated extensive biological and chemical investigations since the 1960s. Early research focused on the identification of the precursors to the well known ancient dye Tyrian purple, and revealed an interesting association between these brominated indole precursors and choline esters. The muscle-relaxant and neurotoxic activity of Muricidae choline esters has been well described in the literature and more recent research has focused on the anticancer properties of the brominated indoles. Despite significant research interest, the ecological and physiological role of the Tyrian purple precursors remains uncertain. However, the combination of biologically active compounds present in *D. orbita* provides interesting potential for nutraceutical development. Increasing biological knowledge on the ecology of the snail, as well as the biodistribution and biosynthesis of secondary metabolites in this species will facilitate sustainable supply. These biological and chemical insights on *D. orbita* provide a good basis for future research and position this species as a suitable model system for novel method development and other innovative research in marine natural product chemistry. 
